# Editorial: Functional and structural brain network construction, representation and application

**DOI:** 10.3389/fnins.2023.1171780

**Published:** 2023-03-10

**Authors:** Weikai Li, Zhengxia Wang, Shuo Hu, Cen Chen, Mingxia Liu

**Affiliations:** ^1^College of Mathematics and Statistics, Chongqing Jiaotong University, Chongqing, China; ^2^School of Computer Science and Technology, Hainan University, Haikou, China; ^3^Department of Nuclear Medicine, Xiangyang Hospital, Central South University, Changsha, China; ^4^Institute for Infocomm Research (A^*^STAR), Singapore, Singapore; ^5^Department of Radiology and Biomedical Research Imaging Center, University of North Carolina at Chapel Hill, Chapel Hill, NC, United States

**Keywords:** functional brain network, morphological network, metabolic network (MBN), network based statistic, high order networks

## Introduction

**Figure 1:**
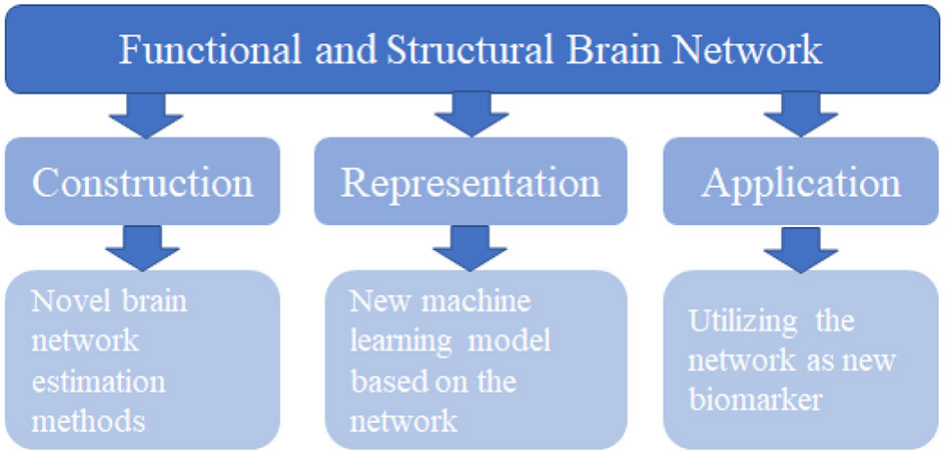
Functional and structural brain network construction, representation, and application.

Structural and functional brain networks have been becoming an increasingly useful tool in understanding the interactions among the separated brain regions, and the pathogenesis of specific neurological disease. In the past decade, there has been an increasing interest in modeling the brain networks based on various mode data (e.g., fMRI, EEG, PET and DTI) and capturing feature representations of brain networks (e.g., connection, graph topology, and graph neural networks) for understanding pathogenesis. Due to the complexity of the brain being far beyond our imagination, revealing the mystery of the brain is still facing many challenges. Thus, there is still debate over the many ways to construct brain networks, how to effectively utilize the multi-modal data, and how to best reveal information about brain health and disorder. The application of network science in the brain has promoted our understanding of structure and functional organization of the brain. Furthermore, studying the brain within this framework effectively reveals how neurological diseases affect brain organization. In this Research Topic, we seek to gather new findings on brain network construction, multimodal fusion, representation of network learning, and making inferences and predictions *via* brain networks. More specifically, the goal of this Research Topic is to promote the current understanding of the brain connectome *via* mathematical modeling, develop new and advanced methods to capture the graphical relationship between function and structure, effectively utilize the multi-modal data, and accurately learn the representation of the network in brain disorders, thereby promoting our understanding of the underlying configuration and dynamics of the brain. From this topic, we can easily find that the main works can be summarized into three categories, i.e., utilizing the network as new biomarker, new machine learning model based on the network, new brain network estimation methods as shown in Figure 1.

## The application of brain network

Here, we firstly review the application of brain network which utilizing the network as new biomarker, the networks can be estimated by fMRI sMRI and even PET. The functional, morphological and metabolic network can be used as a biomarker individually or in a multimodal form.

For the functional networks estimated by fMRI data. Shen et al. focuses on the Chronic ankle instability (CAI) in the views of ALFF, fALFF, ReHo, and FC. CAI not only involves peripheral, but also causes plasticity changes in the central nervous system (CNS). Shen et al. used a data-driven algorithm based on resting-state fMRI (rs-fMRI) to reflect the internal brain activity of CAI patients. Low-frequency fluctuation (ALFF), fractional ALFF (fALFF), and regional homogeneity (ReHo) reveal the consistency of neuronal activity in a region. In addition, functional connectivity (FC) provides functional connectivity between brain regions. The findings suggest that patients with CAI have increased neuronal activity in sensorimotor networks and weakened inter-hemispheric connections in regions of the brain. These results may provide insights into the pathophysiological alteration in CNS among CAI patients. Moreover, Li L. et al. focus on the intra-and inter-network functional connectivity in patients with Crohn's disease. The paper aims to study the interaction mode of resting-state networks (RSNs) as the brain runs as a whole with complex internal networks. The gut-brain axis is the bidirectional communication, which not only maintains gastrointestinal homeostasis, but also affects higher cognitive functions, emotion and motivation through neural pathways. Many studies have reported changes in the structure and function of several brain areas in patients with Crohn's disease (CD). However, little is known about whether the possible functional connectivity of resting-state networks (RSNs) is altered in CD patients. This paper investigates the intra- and inter-network alterations between related RSNs in patients with CD and the potential relationships between altered neuroimaging and CD clinical indices, which provides more insights into the pathophysiological mechanisms of brain plasticity in CD.

For the morphological networks estimated by gray matter data. Peng, Feng et al. used sMRI to construct individual whole-brain morphological networks and further investigated the alterations in the rich-club organization in individuals with SCD as compared with healthy elderly. The morphological networks are estimated by the Jensen-Shannon Divergence Similarity Estimation (JSSE) method (Li et al., 2021). Based on previous findings of disrupted functional and structural connectivity in AD, we hypothesized that altered topological properties of the morphological networks can be detected as early as the SCD stage. Moreover, Yi et al. constructed individual brain networks *via* Jensen-Shannon Divergent Similarity Estimation (JSSE) method for preschool children with ASD and normal controls. The individual brain network indicator based on the JSSE method is an effective indicator for identifying individual-level brain network abnormalities in patients with ASD. The proposed classification method can contribute to the early clinical diagnosis of ASD.

For the metabolic networks estimated by 18F-FDG PET data. The paper by Xue et al. engaged in exploring age-related topological properties and rich-club organization changes. They collected 18F-FDG PET data in the young and aged rats and constructed individual metabolic networks using Jensen-Shannon Divergence Similarity Estimation (JSSE) method. The findings suggested abnormalities in topological properties of individual brain metabolic networks in the aged rats as well as impaired metabolic connectivity in the rich-club organization, providing new insights into age-related brain changes in healthy and diseased rodents. Furthermore, strengthening age-related reorganization mechanism research of the brain network is of great clinical significance for understanding and identifying the function decline and disease progression caused by aging. In addition, Peng, Zhang et al. focus on the rich-club organization of individual brain metabolic networks in parkinson's disease. An individual metabolic connectome based on the standard uptake value (SUV) was built using the Jensen-Shannon Divergence Similarity Estimation (JSSE) method to compare the rich-club properties between PD patients and HC. The results indicated that PD patients had decreased rich club connections but similar feeder and local connections compared with HCs, indicating rich club connections as a promising marker for early diagnosis of PD.

For the multi modal form, Alm et al. examined the independent contributions of structural and functional connectivity markers to individual differences in episodic memory performance in 107 cognitively normal older adults from the BIOCARD study. Structural connectivity, defined by the diffusion tensor imaging (DTI) measure of radial diffusivity (RD), was obtained from two medial temporal lobe white matter tracts: the fornix and hippocampal cingulum, while functional connectivity markers were derived from network-based resting state functional magnetic resonance imaging (rsfMRI) of five large-scale brain networks.

## The representation of brain network

In this section, we briefly review the representation of brain network, which design new machine learning model based on the network.

The first work by Peng, Liu et al. addresses that existing studies have reported the utilization of the information from the connection to train the classifier; such an approach ignores the topological information and, in turn, limits its performance. Specifically, they propose the combination of the information derived from both FBN and its corresponding graph theory measurements to identify and distinguish ASD from normal controls (NCs). Specifically, a multi-kernel support vector machine (MK-SVM) was used to combine multiple types of information.

The second paper by Zhang Y. et al. addresses that the educational level and emotional state of patients, skills and experience of examiners in using MoCA, and the examination environment all affect the cognitive function scores of patients with end-stage renal disease (ESRD). Therefore, an accurate prediction of cognitive function scores plays an important role in subsequent treatment of patients. For this reason, this paper proposed a novel model to explore the relationship between functional magnetic resonance imaging (fMRI) data and clinical scores, thereby predicting cognitive function scores of ESRD patients. It helps to overcome this issue that the scores of the cognitive function are highly subjective, which tend to affect the results of clinical diagnosis for ESRD patients.

The third paper by Li Y. et al. considered the dynamic changes of functional connections in the resting state, they proposed methodology to construct resting state high-order functional hyper-networks (rs-HOFHNs) for patients with depression and normal subjects. Meanwhile, they also introduce a novel property (the shortest path) to extract local features with traditional local properties (cluster coefficients). A subgraph feature-based method was introduced to characterize information relating to global topology. Two features, local features and subgraph features that showed significant differences after feature selection were subjected to multi-kernel learning for feature fusion and classification.

The Graph Neural Network is also an effective technology to utilize the brain network. The fourth paper by Feng et al. propose a novel Deep Spatiotemporal Attention Network (DSTAN) framework for MCI recognition based on brain functional networks. Specifically, they first extract spatiotemporal features between brain functional signals and FBNs by designing a spatiotemporal convolution strategy (ST-CONV). Then, on this basis, they introduce a learned attention mechanism to further capture brain nodes strongly correlated with MCI. Finally, they fuse spatiotemporal features for MCI recognition. The entire network is trained in an end-to-end fashion.

Similarly, Zhang S. et al. provide a novel dense individualized and common connectivity-based cortical landmarks (DICCCOL)-based multi-modality graph neural networks (DM-GNN) framework to differentiate preterm and term infant brains and characterize the corresponding biomarkers. In practice, the functional magnetic resonance imaging (fMRI) of the brain provides the features for the graph nodes, and brain fiber connectivity is utilized as the structural representation of the graph edges. Self-attention graph pooling (SAGPOOL)-based GNN is then applied to jointly study the function and structure of the brain and identify the biomarkers.

## The construction of brain network

In this section, we briefly review the construction of brain network, which design new brain network estimation methods.

In contrast to the group-level network methods for morphological network modeling, Xu et al. provide an individual morphological network estimation method by Jensen-Shannon Divergent Similarity Estimation (JSSE). The graph metrics are then calculated. In the end, the multiple kernel support vector machine (MK-SVM) was used for combining brain connectomes and graph metrics for differentiating SCD from NCs.

Further, the machine learning trick is also adopted in brain network estimation. Jiang et al. focus on correlation's correlation (CC), for constructing the high-order brain network. Besides, for understanding CC more rigorously and providing a systematic brain network learning framework, they reformulate it in the Bayesian view with a prior of matrix-variate normal distribution.

## Conclusion

All papers tackle different but extremely relevant domains of the functional and structural brain network construction, representation and application. We believe this topic will raise awareness in the scientific community, through presenting and highlighting the advances and latest novel and emergent technologies, implementations, applications concerning the brain network. In closing, we would like to thank all the authors who submitted their research work to this special issue. We would also like to acknowledge the contribution of many experts in the field who have participated in the review process and provided helpful suggestions to the authors to improve the contents and presentations of the articles.

## Author contributions

All authors listed have made a substantial, direct, and intellectual contribution to the work and approved it for publication.
